# A Rare Case of Prototheca Algaemia in a Patient with Systemic Lupus Erythematosus and Recent Belimumab Infusion

**DOI:** 10.1155/2012/754901

**Published:** 2012-11-21

**Authors:** Carolina Mejia-Otero, Shelley Singh, Luis Arias Urdaneta, Carlos Sesin, Anindita Chakrabarti, Nanci Mae Miller, Claudio Tuda

**Affiliations:** ^1^Internal Medicine Department, Mount Sinai Medical Center, Miami, FL 33140, USA; ^2^Division of Rheumatology, Internal Medicine Department, Mount Sinai Medical Center, Miami, FL, USA; ^3^Department of Infectious Disease, University of Miami Miller School of Medicine, Miami, FL 33136, USA; ^4^Department of Pathology, Mount Sinai Medical Center, Miami, FL, USA; ^5^Division of Infectious Disease, Internal Medicine Department, Mount Sinai Medical Center, Miami, FL, USA

## Abstract

Novel agents for the treatment of immune-mediated diseases such as systemic lupus erythematosus (SLE) have been increasingly used as an alternative to or in combination with conventional therapies. Belimumab, a human monoclonal antibody that inhibits B-cell activating factor (BAFF), has demonstrated efficacy in moderate-to-severe SLE with similar adverse effects when compared to other biologic agents and conventional SLE therapies. Here, we describe a woman with SLE and diabetes mellitus (DM) on immunosuppressive therapy for five years who was admitted to the hospital for pneumonia but had a complicated hospital course with multiple infections and, most notably, a nosocomial algaemia due to *Prototheca wickerhamii*, which was treated successfully with amphotericin B. She had recently received three belimumab infusions as an outpatient prior to admission to the hospital. To the best of our knowledge no cases of human protothecosis in patients receiving belimumab have been described in the English literature; however, unusual infections have to be considered in all patients undergoing immunosuppressive therapies who persist with fever despite conventional antimicrobials.

## 1. Introduction

 SLE is a heterogeneous disease caused by an aberrant autoimmune response that spares no organ and affects people of African, Hispanic, and Asian ancestry more than other racial or ethnic groups [1, 2]. Genetic, environmental, hormonal, and immunoregulatory factors contribute to the expression of tissue injury and clinical manifestations. Both T and B cell antigen receptor-mediated activation are altered and early signaling events are amplified [1, 3]. All B-cell subgroups contribute to the production of autoantibodies. These play an important role in the presentation of antigens and autoantigens to T cells, thus mediating tissue damage and contributing to disease expression [1].

 A better understanding of the pathogenesis of immune-mediated diseases has led to the development of a new therapeutic approach to SLE, B-cell-targeted therapy. This acts through two principal mechanisms: direct killing by monoclonal antibodies specific for B-cell surface molecules CD19, CD20 (rituximab, ocrelizumab), and CD22 (epratuzumab) and attrition due to the inhibition of B-cell survival factors BLyS (belimumab) and APRIL (atacicept) [4]. Belimumab is the first targeted biological treatment that is FDA approved specifically for the treatment of SLE [5]. Previous randomized controlled trials have shown reductions in disease activity and prevention of flares [6, 7], with an acceptable safety profile [8]; however, malignancies, serious or severe infections, and infusion reactions have been described [4, 5]. A case of algaemia with *P. wickerhamii* after belimumab infusion is reported here.

## 2. Case Presentation

 We describe a case of a 67-year-old female with SLE on immunosuppressive therapy for more than five years who presented to the hospital with a two-day history of cough, fever, and fatigue. One week prior to admission, she received her third belimumab infusion (10 mg/kg) without acute complications. Her first two loading doses of belimumab were four and two weeks earlier, again, without incident. The patient was diagnosed with SLE over five years ago, based upon a history of immune thrombocytopenia and autoimmune hemolytic anemia (Evan's syndrome), hypocomplementemia, polyarthralgias, and a positive ANA in a 1 : 160 speckled pattern. She had multiple flares of hemolytic anemia requiring high doses of steroids as well as several immunosuppressive therapies. Her regimen at the time of her admission consisted of azathioprine 50 mg twice daily and prednisone 60 mg daily. Her comorbidities included DM, essential hypertension, drug-induced osteoporosis, and cataracts, as well as a history of a left middle cerebral artery aneurysm status after clipping. Other medications were long-acting insulin, tramadol, lisinopril, metoprolol, and folic acid.

**Figure 1:**
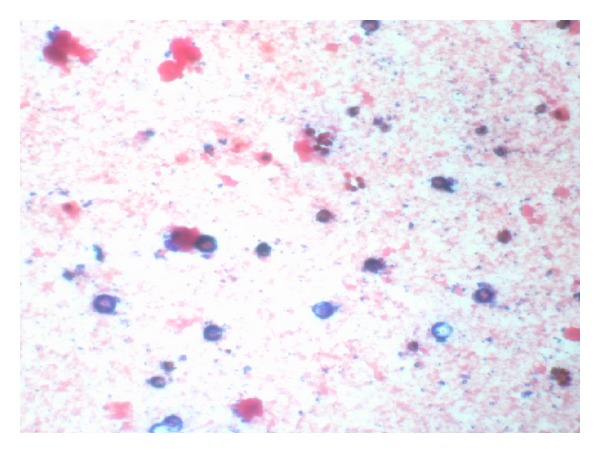
Gram stain of yeast-like colonies from blood and chocolate agar showing large gram-positive spherical cells of varied sizes from 8 uM to 24 uM in diameter. *Courtesy of the Department of Pathology-Microbiology at Mount Sinai Medical Center of Florida. *

**Figure 2:**
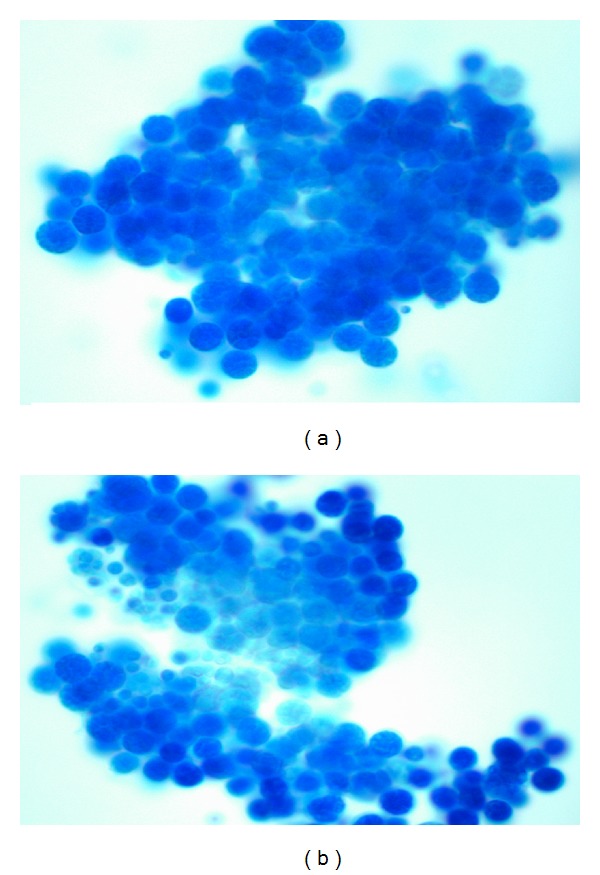
Yeast-like cells of variable sizes resembling endospores contained in a sporangiospore seen on a wet mount prepared using lactophenol cotton blue, reported as *Prototheca* based on the morphology of the organism. *Courtesy of the Department of Pathology-Microbiology at Mount Sinai Medical Center of Florida. *

 At admission, the patient was tachycardic with a low-grade fever and hypoxemia. Physical examination revealed coarse breath sounds bilaterally. Laboratory data and imaging showed leukocytosis and bilateral infiltrates consistent with multifocal pneumonia. IV antibiotics were initiated; however, the patient subsequently developed respiratory failure leading to multiple intubations throughout her hospital course and eventual tracheostomy. Results of bronchoalveolar lavages revealed *Pneumocystis jiroveci*, *Cytomegalovirus*, and *Herpes simplex virus*; blood cultures grew *E. faecalis* and *K. pneumoniae* (see Table 1). Multiple antimicrobials were employed to treat the numerous infections, including clindamycin, gancyclovir, linezolid, ceftaroline, ampicillin, valacyclovir, and trimethoprim-sulfa. The patient had persistent fevers despite multiple antimicrobial therapies and subsequent blood cultures yielded the growth of P. wickerhamii (Figures 1 and 2), which was treated with amphotericin B for two weeks. Repeat blood cultures remained negative after treatment.

## 3. Discussion

 Human protothecosis is a rare infection caused by members of the genus *Prototheca*, a microscopic single-celled heterotrophic, achlorophyllic algae that belongs to the family Chlorellaceae. It reproduces by endosporulation and binary fission and is ubiquitous in nature; the most common species causing infection in humans is *P. wickerhamii* [9–11]. The appearance of *Prototheca* is similar to yeast on routine media but may be distinguished from yeast on wet mounts with lactophenol cotton blue staining if typical morula forms are observed [12].

 Patients at risk for developing protothecosis are those with chronic steroid use, hematological or solid tissue malignancy, or other immunocompromised states such as DM, autoimmune disease, or primary immunodeficiency; however, cases in immunocompetent patients have also been described [13]. In the immunosuppressed individual, opportunistic infection with *Prototheca* species may be associated with bacterial, viral, or fungal coinfection, complicating both diagnosis and treatment [12]. 

 Cellular immunity and polymorphonuclear leukocytes (PMNs), along with IgG antibodies and serum opsonins, are involved in the host defense against *Prototheca spp.* Optimal phagocytosis and killing of *P. wickerhamii* by PMNs require the presence of both specific IgG antibody and heat-labile opsonins observed after ultrastructural studies [14]. 

To date, there are 160 reported cases of human protothecosis, six (4%) of which have documented algaemia [13]. Most *Prototheca* infections occur on the skin or bursae; blood infections are rare and are highly associated with an immunocompromised state, such as that which occurred with this patient with SLE, DM, and on immunosuppressive therapy. Nonetheless, only after receiving belimumab did she develop protothecosis. We propose that belimumab may have contributed to the development of this *Prototheca* infection, as her longstanding immunosuppressed state had not led her to this rare opportunistic infection in the past.

One case report of protothecosis in a hematopoietic stem cell transplant recipient has been described after infliximab treatment, another biologic agent that can lead to immunosuppression [9, 15]. No cases of human protothecosis in patients receiving belimumab have been described in the literature thus far, but the correlation does not necessarily mean causation in our patient since she was on corticosteroids and azathioprine prior to the first dose of belimumab. Nevertheless, this paper illustrates that unusual infections have to be considered in all patients undergoing immunosuppressive therapy who persist with fever despite conventional antibiotic, antiviral, and antifungal treatments. Further studies or observation of uncommon adverse events in patients with belimumab are needed.

## Figures and Tables

**Table 1:** Positive microbiology cultures during hospitalization.

Date	Source of culture	Organism
5/27/12	Urine	*Escherichia coli *
5/31/12	Bronchoalveolar lavage	*Cytomegalovirus *
6/1/12	Blood	*Cytomegalovirus *
6/12/12	Bronchoalveolar lavage	*Pneumocystis jiroveci *
7/3/12	Bronchoalveolar lavage	*Herpes simplex virus *
7/9/12	Blood	*Cytomegalovirus *
7/15/12	Urine	*Yeast, Enterococcus species *
7/15/12	Blood	*Enterococcus faecalis *
7/19/12	Urine	*Yeast, Enterococcus species *
7/19/12	Blood	*Enterococcus faecalis*, *Klebsiella pneumonia *
7/20/12	Blood	*Prototheca wickerhamii*, *Klebsiella pneumonia *
